# How we read pediatric PET/CT: indications and strategies for image acquisition, interpretation and reporting

**DOI:** 10.1186/s40644-017-0130-8

**Published:** 2017-11-07

**Authors:** Gabrielle C. Colleran, Neha Kwatra, Leah Oberg, Frederick D. Grant, Laura Drubach, Michael J. Callahan, Robert D. MacDougall, Frederic H. Fahey, Stephan D. Voss

**Affiliations:** Department of Radiology, Boston Children’s Hospital, Harvard Medical School, 300 Longwood Avenue, Boston, MA 02115 USA

**Keywords:** PET/CT, Diagnostic CT, Pediatric oncology, Hybrid imaging, Dose reduction, Attenuation correction, Multidisciplinary interpretation

## Abstract

PET/CT plays an important role in the diagnosis, staging and management of many pediatric malignancies. The techniques for performing PET/CT examinations in children have evolved, with increasing attention focused on reducing patient exposure to ionizing radiation dose whenever possible and minimizing scan duration and sedation times, with a goal toward optimizing the overall patient experience.

This review outlines our approach to performing PET/CT, including a discussion of the indications for a PET/CT exam, approaches for optimizing the exam protocol, and a review of different approaches for acquiring the CT portion of the PET/CT exam. Strategies for PACS integration, image display, interpretation and reporting are also provided.

Most practices will develop a strategy for performing PET/CT that best meets their respective needs. The purpose of this article is to provide a comprehensive overview for radiologists who are new to pediatric PET/CT, and also to provide experienced PET/CT practitioners with an update on state-of-the art CT techniques that we have incorporated into our protocols and that have enabled us to make considerable improvements to our PET/CT practice.

## Introduction

Positron Emission Tomography/Computed Tomography (PET/CT) plays an important role in the diagnosis, staging and management of a wide range of pediatric malignancies including Hodgkin and non-Hodgkin lymphoma, malignant soft tissue and bone sarcomas, head and neck tumors, Langerhans cell histiocytosis (LCH) and neuroblastoma [1–4]. PET/CT is the most common hybrid imaging technique currently in use, owing to the increased sensitivity and specificity of PET/CT for detecting metabolically active malignancies. ^18^Fluorine-2-fluoro-2-deoxy-d-glucose (FDG) is still the most commonly used radiopharmaceutical in routine clinical use, but a number of new PET tracers are being developed with potential for use in imaging children with cancer.

In addition to the discovery and development of novel radiopharmaceuticals for detection of malignant disease, the techniques used to acquire PET images have also evolved. PET imaging was initially confined to review of emission imaging data. Non-diagnostic quality transmission scans were obtained only for soft tissue attenuation correction of the PET raw data, but provided no additional anatomic information. With the development of integrated hybrid PET/CT scanners, many new opportunities emerged for obtaining high quality co-registered CT images [5]. Because we now have numerous options for obtaining CT images as part of the PET/CT acquisition there is an increasing need for awareness and selection of appropriate CT imaging techniques. These CT techniques may vary substantially, and are largely dependent on whether the CT images are intended for attenuation correction only, anatomic co-localization, or diagnostic interpretation [6].

This review will outline our approach to performing PET/CT in children with a variety of pediatric cancers. We will review current indications and common practices for using PET/CT and the evidence supporting these practices, and discuss the practical aspects of performing and interpreting PET/CT examinations in children.

## Indications

**Fig. 1:**
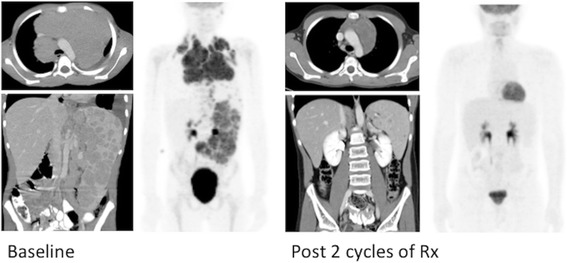
PET/CT in Hodgkin Lymphoma. Baseline PET/CT shows bulky mediastinal, extensive splenic and intra-abdominal nodal disease. Following 2 cycles of chemotherapy the ^18^F-FDG PET/CT shows a complete metabolic response to therapy, whereas residual mediastinal soft tissue mass and splenic lesions remain. Following a response-based treatment algorithm, subsequent therapeutic decisions are made based on the metabolic complete response (CR) shown by PET/CT

Indications for performing (and reimbursing) PET/CT differ between countries and healthcare systems. In the US, reimbursement programs also differ between states and between insurance companies. While private insurers may exercise more latitude in considering payment for imaging pediatric cancers, most third party payers initially rely on the national coverage determinations provided by the Centers for Medicare and Medicaid Services (CMS) for guidance. CMS has expanded the reimbursable indications for use of PET/CT to include the majority of adult cancers [7]. In some cases there is overlap with pediatric cancers, such as lymphoma and melanoma, although for the majority of pediatric malignancies CMS does not provide explicit guidance or approval. As such, in most pediatric cancer centers such as ours, insurance pre-authorization is sought for nearly all new cancer diagnoses prior to obtaining a PET/CT, whether for initial staging or for interim response assessment. CMS approved indications, for which specific reimbursement codes exist, include both Hodgkin and non-Hodgkin lymphoma, melanoma, neuroendocrine tumors, and Langerhans Cell Histiocytosis, as well as other malignancies more commonly seen in adults but occasionally occurring in children (e.g. colorectal and esophageal cancer). In the case of Hodgkin lymphoma, the use of FDG PET/CT to evaluate early metabolic response to therapy has led to new response-based treatment algorithms and completely changed the approach therapy (Fig. 1) [3, 8].

In many other pediatric cancers, including Ewing sarcoma, rhabdomyosarcoma, synovial cell sarcoma, osteosarcoma, gastrointestinal stromal tumor (GIST) and MPNST, there is accumulating data showing the importance of PET/CT in staging, and in some cases response assessment. For the staging of osteosarcoma, Ewing sarcoma and rhabdomyosaroma there is consistent evidence of improved sensitivity of PET/CT for detecting skeletal metastases when compared to conventional techniques such as bone scintigraphy [2]. The data is less clear regarding the utility of PET/CT for assessing response and predicting outcome in the malignant sarcomas, although several small studies have shown a correlation between changes in FDG uptake (standardized uptake value, SUV) and response to therapy and outcome [9–11]. Pediatric malignancies are comparatively rare, making it difficult to design large clinical trials to demonstrate the utility of FDG PET/CT in the management of most pediatric cancers. As of yet no prospective trials have incorporated response-based treatment decisions into algorithms that rely solely on changes in FDG uptake to dictate course of therapy, which limits our assessment of the prognostic value of PET in these malignancies.

In neurofibromatosis type-1, FDG uptake is an effective biomarker for predicting evolution of benign neurofibromas into either premalignant atypical neurofibromas or malignant peripheral nerve sheath tumors (MPNST) [12]. Many other pediatric cancers have been shown to be FDG-avid and a staging PET/CT has been shown in many small studies to have improved sensitivity over existing techniques [4, 13, 14]. In contrast, neuroblastoma, despite being the most common non-CNS solid tumor occurring in children, is not routinely imaged by FDG PET/CT, owing mostly to the large body of evidence showing the value of ^123^I-MIBG for staging and predicting outcome after response to induction therapy, in addition to establishing the extent of disease prior to beginning treatment with ^131^I-MIBG [15, 16]. In addition, in many cases neuroblastoma is FDG-negative and as such the routine use of FDG PET in staging and response assessment in neuroblastoma has been largely restricted to those few patients with MIBG-negative disease [17]. Wilms tumor is the most common renal tumor occurring in childhood, although physiologic excretion of FDG from the kidneys has limited the routine use of PET/CT for management of Wilms tumor [18]. In our experience, however, PET imaging may still be useful for staging, particularly for characterizing extrarenal sites of disease, and for re-staging at the time of relapse.

**Fig. 2:**
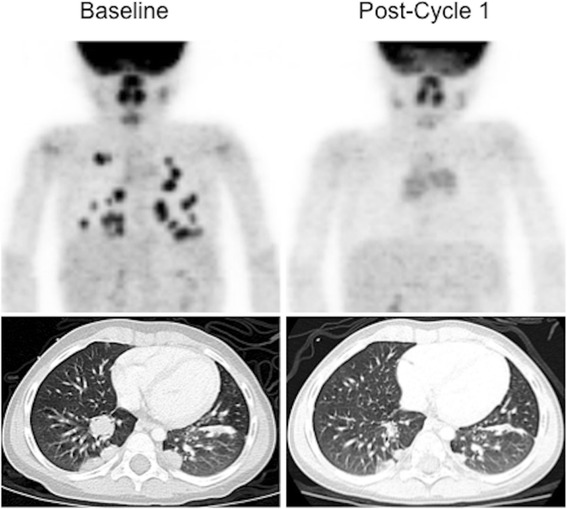
PET response to targeted experimental Phase 1 therapy: Crizotinib in ALK^+^ IMFT. ^18^F-FDG-PET and CT imaging of Crizotinib response in IMFT: Baseline whole body ^18^F-FDG-PET and Chest CT show multiple FDG-avid pulmonary nodules, confirmed by biopsy to be IMFT. Following 1 cycle of therapy, no residual abnormal FDG accumulation is seen (metabolic CR). The lesions have decreased significantly in size by CT, meeting criteria for partial reponse (PR), but not CR. This patient has remained free of disease for >36 months [32].

Unique molecular targets are also being identified for many pediatric malignancies. Tumors such as inflammatory myofibroblastic tumor (IMFT), as well as other uncommon pediatric neoplasms, are receiving renewed attention based on the identification of molecular markers against which targeted therapies can be directed. The use of FDG PET/CT to monitor changes in tumor metabolic activity in response to treatment with molecularly targeted agents, may be an important surrogate for establishing pharmacologic activity of new drugs being evaluated in early phase clinical trials [19]. In particular, alterations in tumor metabolic activity can play an important role in guiding therapy, even in when significant measureable changes in lesion size and/or number are not observed (Fig. 2).

## Practical aspects of PET/CT: ordering, protocoling, acquiring, and interpreting the PET/CT examination

### Ordering a PET/CT

PET/CT is typically performed as either an examination of the whole body or the torso (eyes-to-thighs), although more focused limited examinations may be specified by the ordering physician. In general, lymphoma patients undergo examinations of the torso, which assures coverage of the majority of the lymphoid tissue, extending from Waldeyer’s ring through the inguinal lymph nodes. Because of the potential for metastatic disease occurring anywhere in the body, sarcoma patients usually undergo a whole-body PET/CT. For other patients the extent of coverage should be determined in conjunction with the ordering clinician to ensure adequate coverage of specific regions of interest based on the patient’s disease, and to be in compliance with coverage determined at the time of insurance pre-authorization. Some third party payers will approve a PET/CT of the torso, but deny coverage for a whole-body exam.

**Fig. 3:**
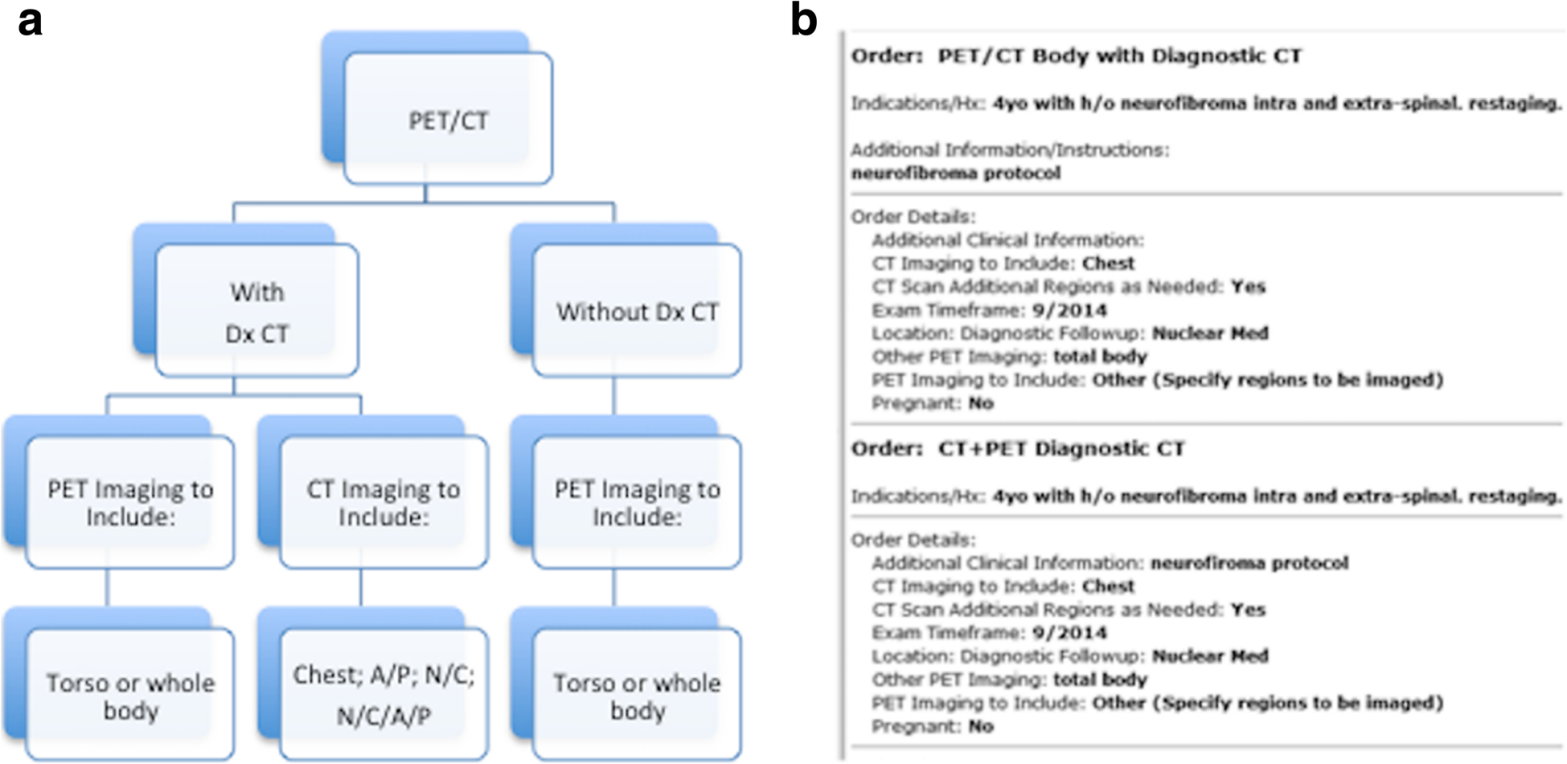
Guide to ordering a PET/CT together with diagnostic CT imaging. An algorithmic approach (**a**) to ordering a PET/CT exam provides clinicians with prompts for specific information that guides the ordering process and encourages an integrated approach to protocoling both the PET/CT and the diagnostic CT exams. The subsequent order (**b**) contains the necessary clinical information to allow both the PET/CT and the diagnostic CT examinations to be correctly protocoled

At the time a PET/CT study is ordered, the referring clinician should determine if a diagnostic quality CT (Dx CT) is required or whether a low-dose CT for anatomical correlation will suffice [20]. To aid clinicians in ordering the correct examination, an algorithmic approach is useful (Fig. 3). If a diagnostic study is required, the necessary extent of body coverage must be clearly stated by the referring physician. Exam techniques and parameters must then be clearly delineated by the protocolling radiologist. For example, if a diagnostic abdomen and pelvis examination is required, the Dx CT should be protocoled with intravenous and oral contrast media, using established departmental guidelines. The same is true for a Dx CT of the entire torso (Neck/Chest/Abdomen and Pelvis). If only a diagnostic chest CT is required, a determination must be made whether the CT chest is for the purpose of characterizing mediastinal adenopathy and soft tissue disease, in which case IV contrast media is required, or for the identification and characterization of pulmonary nodules. In this latter instance, a non-contrast protocol is typically used and the examination is performed at end-inspiration to optimize visualization of small lung nodules.

**Fig. 4:**
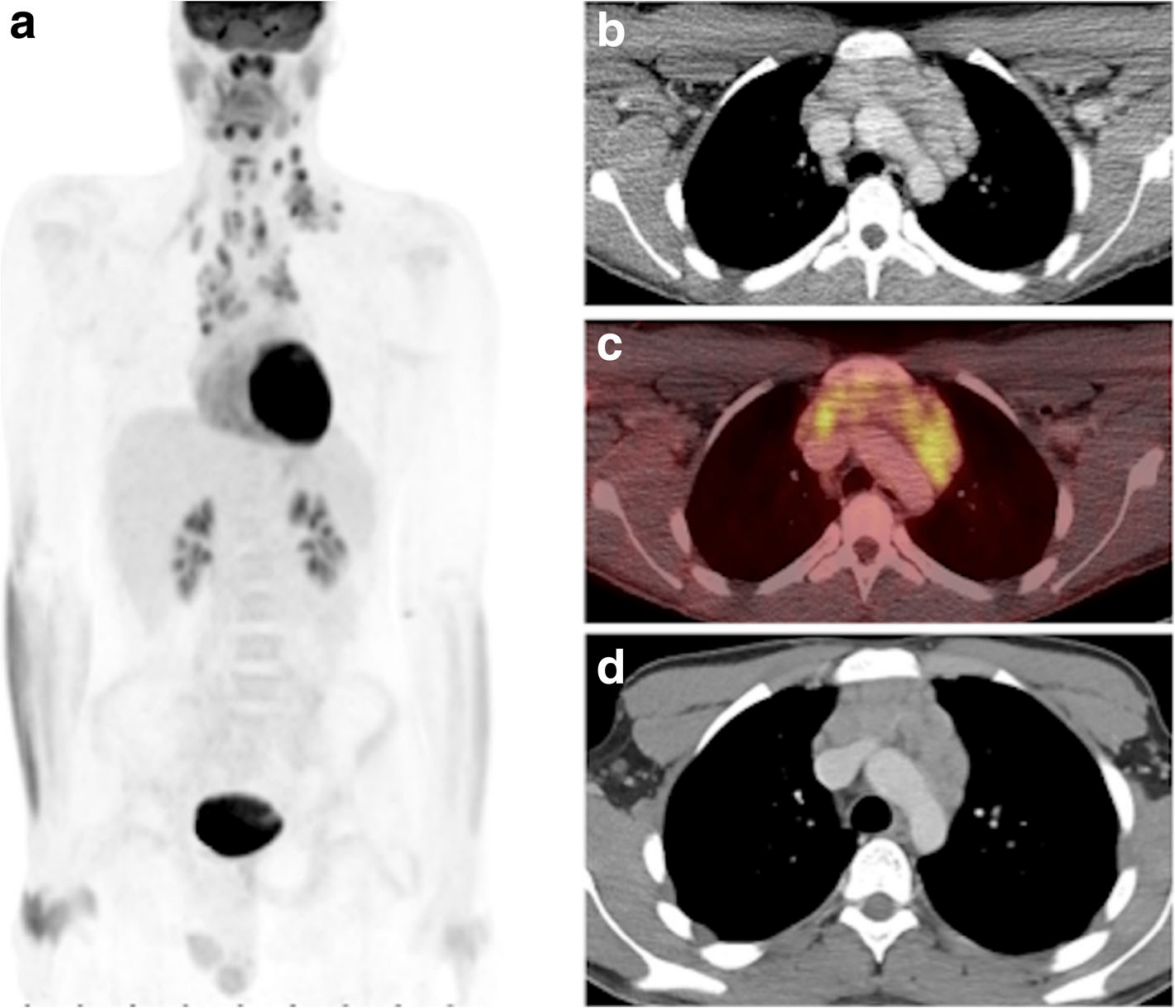
Contrast enhanced low dose attenuation correction CT from PET/CT exam compared to diagnostic CT. The low dose attenuation correction CT, performed with IV contrast (**b**, **c**) provides anatomic localization of the PET findings (**a**), and – while not of diagnostic quality – is comparable to the diagnostic CT (**d**), and has sufficient diagnostic information to warrant a thorough review

**Fig. 5:**
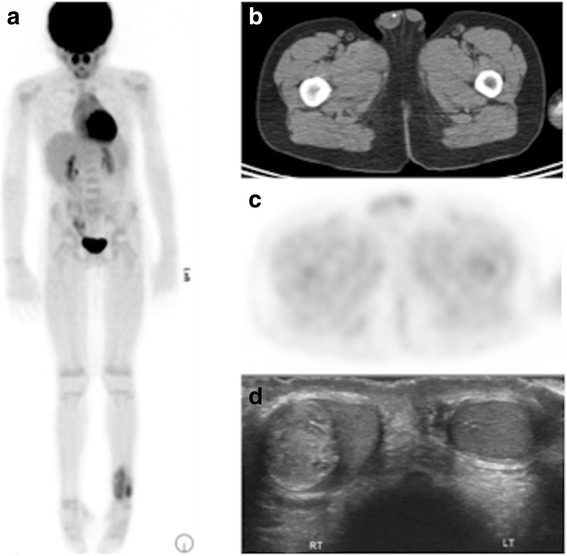
The attenuation correct CT has diagnostic value. Eight years old patient with Ewing sarcoma of the left distal tibia. ^18^F-FDG PET/CT shows uptake in the primary tumor, but no metastatic disease (**a**). Scrotal calcification was detected incidentally on the AC CT (**b**), but was not associated with FDG uptake (**c**). Ultrasound confirmed a mass (**d**), which was revealed to be a mature teratoma, unrelated to the primary tumor

If a Dx CT is not required, the CT portion of the PET/CT examination is protocoled using the lower dose attenuation correction CT (AC CT, lowest dose) protocol. Many departments distinguish between an AC CT and an anatomic co-localization CT (slightly higher CT dose, but not as high as a diagnostic CT), and a diagnostic quality CT. We only have a single AC CT protocol, with slighter higher tube current for patients > 55 kg, as compared to smaller patients < 55 kg (see later), and routinely utilize IV contrast for the AC CT, to improve anatomic localization and image quality despite the lower CT dose. Importantly, referring clinicians should understand that the low dose AC CT, while not considered to be of diagnostic quality, nonetheless contains images that in many instances are comparable to diagnostic scans (Fig. 4). As such, it is our practice to routinely issue a separate report summarizing any pertinent findings detected on the AC CT. This assures a rigorous review of all the patient’s imaging data, and in some instances, may identify an unexpected finding that alters patient management (Fig. 5), which is consistent with reported rates of 3-5% for clinically significant incidental findings identified on AC CT images [21].

### Patient preparation

Patients being sedated for the PET/CT have nothing by mouth (NPO) after midnight the night before the study. For studies being performed without sedation, patients should be NPO at least 4 h before the exam. In all cases the patients remain fasting during the 1-h FDG uptake period. Because insulin secretion is stimulated by caloric intake, high levels of FDG uptake can occur in both skeletal and cardiac muscle, limiting the interpretability of the PET scan. NG tube feeds, parenteral nutrition, and all dextrose-containing IV solutions should also be discontinued. If IV hydration is required a simple isotonic solution of normal saline is preferred. Note that even non-caloric artificial sweeteners such as Nutrasweet® can stimulate insulin secretion and are contraindicated prior to performing FDG PET/CT [22]. Strenuous exercise is also avoided for 24 h prior to the examination to minimize background uptake in skeletal muscle.

The possibility of pregnancy must be considered in all post-pubertal females. Even though the likelihood of pregnancy is very small in oncology patients due to ongoing chemotherapy and the underlying oncologic illness, most institutions have a policy that requires anyone undergoing a CT examination (and by extension a PET/CT examination) to have a negative pregnancy test prior to the procedure. In our institution, all post-menarcheal female patients age 12 and older undergo urine pregnancy testing prior to the PET/CT exam only if they are also having a diagnostic CT of the abdomen or pelvis; pregnancy tests are not obtained for routine PET/CT studies. The practical and ethical considerations that attend this policy, particularly regarding patient confidentiality for older teenage patients and procedures for responding to a positive pregnancy test, are beyond the scope of this review and must be individualized for each institution.

Diabetic patients require special instructions when preparing for an FDG PET/CT examination [22]. Having a skilled nursing team available to communicate with the patient and family several days prior to arrival will help to avoid either a cancelled examination or an un-interpretable study. Diabetic patients (whether type 1 or 2) can have high circulating glucose levels, either due to absence of insulin production (type 1) or insulin resistance (type 2). Ideally diabetic patients should be scheduled early in the morning. Type 1 patients require exogenous insulin to maintain basal levels of insulin, even while fasting. The long acting form of insulin (NPH), when given around bedtime the prior evening, should be sufficient to maintain appropriate insulin levels until the examination is complete. Blood glucose levels must be checked prior to tracer injection. If higher than 200 mg/dl the examination should probably not be performed as high serum glucose levels can compete with the trace amounts of FDG being administered, thereby reducing the sensitivity of the PET/CT examination.

Short acting insulin should not be administered prior to, or during the PET examination as insulin-mediated muscle uptake will occur, and may limit interpretability of the scan. Many patients with type 1 diabetes mellitus use an insulin pump, and any alteration in the insulin-pump regimen should be made in consultation with the clinician who routinely helps the patient and family manage the insulin pump. Although type 2 diabetes is less common in children, its prevalence is increasing in pediatric populations. Patients with type 2 diabetes mellitus may be treated with metformin, which is associated with undesirable colonic, hepatic and muscle uptake of FDG. Ideally, metformin is discontinued for at least 2-3 days prior to the FDG PET examination, although this may not be possible if it will result in unacceptable hyperglycemia. In rare instances it may be necessary to suppress myocardial FDG uptake (e.g. cardiac and pericardial tumors). This can be accomplished by restricting the patient to a high-fat ketogenic diet, although in practice we use this technique primarily for non-oncologic cardiac imaging applications.

For patients who are having a Dx CT as part of their PET/CT examination, special care must be taken when administering IV and oral contrast agents, to ensure the high diagnostic quality of the CT examination. To the interpreting radiologist, a Dx CT that is obtained on the PET/CT scanner as part of the PET examination should be equivalent in quality to a CT performed on a comparably equipped standalone CT scanner. For oral contrast media, we routinely use between 90 and 300 ml (adjusted by patient age) of contrast, prepared as a 1:30 solution of non-ionic iodinated contrast (Optiray 320, Ioversol, Liebel-Flarsheim Company LLC, Raleigh, NC) diluted into water: for example a 10 yo child will receive 180 ml of contrast, prepared as 6 ml of Optiray 320 diluted into 180 ml water. Although many children may prefer contrast diluted in juice, most juices contain sweeteners and should be avoided. In practice, the non-ionic contrast, when diluted into water, is tasteless and generally well-tolerated. Most dilute barium preparations (BaroCAT) and other palatable oral contrast media contain sweeteners and should be avoided.

### Sedation

For patients requiring sedation, additional direct communication with the sedation or anesthesia team should take place prior to performing the PET/CT examination. This will ensure that any fasting or feeding requirements, and the administration of enteric contrast, is in accordance with anesthesia guidelines. We generally wait approximately 60 min after the last administration of enteric contrast before sedating a patient, thereby reducing the risk of an aspiration event that can occur in a patient with a full stomach. The sedation team also should be aware of the need for all IV fluids, including IV medications in saline solutions, to be glucose/dextrose-free.

Sedated patients typically receive continuous IV fluids both prior to and during the examination. As a result, the bladder may become quite full and in small children, as well as in children with pelvic neoplasms, excreted FDG in a very full bladder can obscure areas of interest and concern, and thereby compromise the examination. Placement of a bladder catheter is ideally done after consultation with the referring oncology team, although in practice catheter placement can be accomplished quickly and without incident. In neutropenic patients, who are at increased risk for development of infection, and in patients with hemorrhagic cystitis, placement of a bladder catheter may be contraindicated, and the decision to place a catheter should be made only after conferring with the ordering oncologist.

### PET/CT acquisition

Although this review is focused on PET/CT, it should be noted that PET/MR has been shown to result in substantial reductions in radiation dose to the patient, primarily due to the elimination of the CT component of the PET/CT [23, 24]. Increased sensitivity of newer generation solid state PET detectors and longer PET acquisition times during the PET/MR exam can further contribute to dose reduction by allowing for lower administered activities of PET radiopharmaceutical. In some instances PET/MR may be preferable, particularly when anatomic co-localization of disease evident on MRI cannot be accomplished by CT [25]. In almost all cases, assuming a portion of the MRI is being performed for diagnostic purposes and not simply for attenuation correction, the combined PET/MR examination will require considerably more time (up to three times longer, depending on the protocol) than a PET/CT providing comparable coverage.

We have recently published a survey of 19 North American institutions where a large volume of pediatric PET/CT examinations are performed [5]. There was surprising variability in practice between institutions, particularly when the PET/CT was ordered together with a diagnostic CT. What follows is a description of the approach developed at Boston Children’s Hospital for performance of PET/CT, with an aim toward optimizing the scanning technique to avoid duplicate CT scanning over the same coverage area and to provide improved anatomic detail when needed to correlate with findings on the accompanying FDG PET scan.

#### Attenuation correction CT

**Fig. 6:**
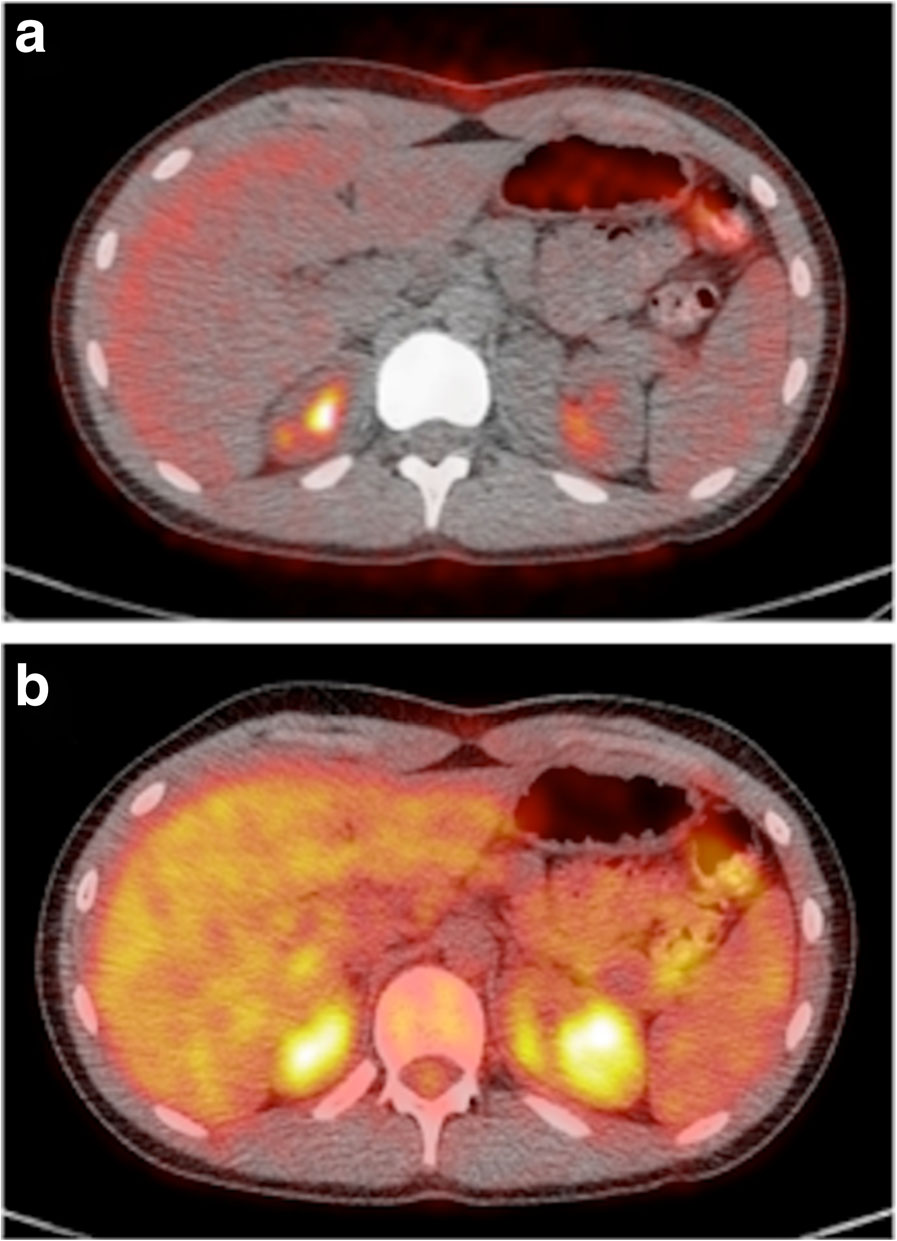
CT based attenuation correction of PET images. Low dose attenuation correction CT exams are not generally used for routine diagnostic interpretation. The AC CT accounts for differences in location/depth from which the 511 keV PET photons are emitted, and can be used to correct for differences in surrounding tissue density (HU density) and the degree to which those tissues absorb or attenuate the PET photons [5]. **a** shows the fused, uncorrected PET data, with increased signal at the periphery of the image and poor signal from middle of the torso and adjacent to the vertebral column. The attenuation corrected image of the same PET data (**b**) provides a more accurate representation of the ^18^F-FDG uptake

The attenuation correction CT portion of the PET/CT exam is usually obtained prior to the PET acquisition. Attenuation correction accounts for differences in the location of positron annihilation events and the degree to which tissues “attenuate” the PET annihilation photons (Fig. 6). Photons emitted from the center of the patient must pass through more attenuating tissue, and thus are less likely to be detected than those from the periphery. Furthermore, photons emitted from tissues with minimal attenuation (i.e. air/lung) are more likely to reach the detector than photons emitted from locations within or near dense structures like bone. A more extensive discussion of the physics underlying the use of CT for PET attenuation correction is beyond the scope of this review and can be found in the recent review by Fahey et al., and references therein [5].

**Fig. 7:**
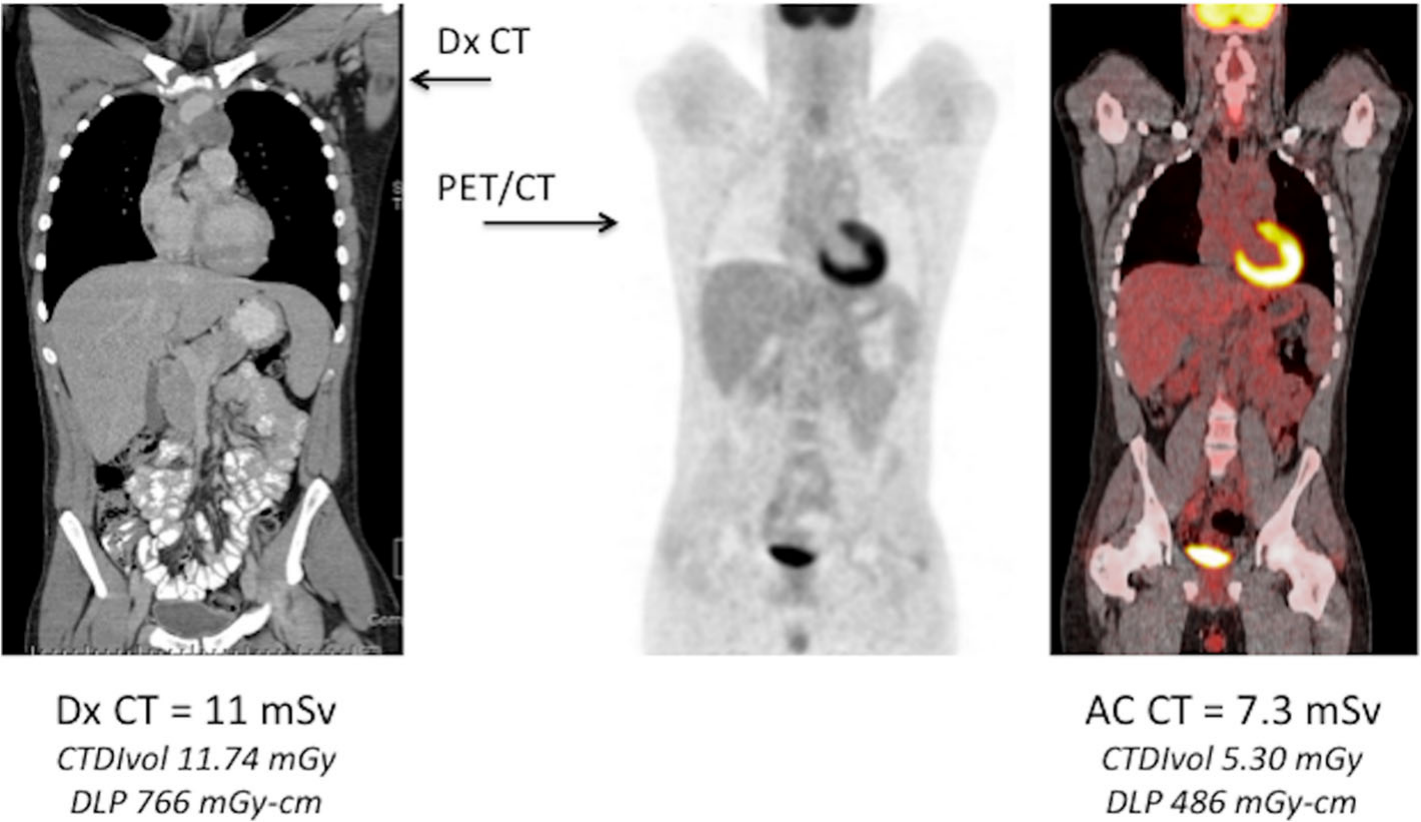
The Double Torso Exam: PET/CT + Diagnostic CT. Dx CT and PET/CT exams performed on the same patient with Hodgkin lymphoma. Both examinations were performed during the same appointment, following 2 cycles of chemotherapy, and show similar coverage between the two CT exams. In this example, the AC CT dose is approximately 2/3 of the Dx CT dose. Eliminating one of the two CT exams and performing a single CT, offers an opportunity for significant CT dose reduction

Traditionally, the CT component of a torso PET/CT exam had been performed at our institution as a low-dose non-diagnostic scan using one of two weight-based low dose CT protocols. When a Dx CT was also requested, this frequently resulted in duplicate imaging of the same coverage area, with as much as 25-50% additional CT dose (Fig. 7). By integrating the non-Dx and the Dx CT data there was an opportunity to eliminate duplicate scanning over regions where both CT’s were being acquired. The Dx CT provides diagnostic quality data for anatomic correlation and can also be used for attenuation correction [20]. However, two concerns were frequently expressed: 1) IV contrast media used for diagnostic CT changes tissue attenuation and thereby might affect the attenuation corrected PET data and SUV calculations, and 2) existing software only allowed for a single series CT acquisition during the PET/CT. If just a Dx Abdomen/Pelvis CT was required, there was no means of merging the Dx CT and low dose AC CT data in a single reconstructed data set.

**Fig. 8:**
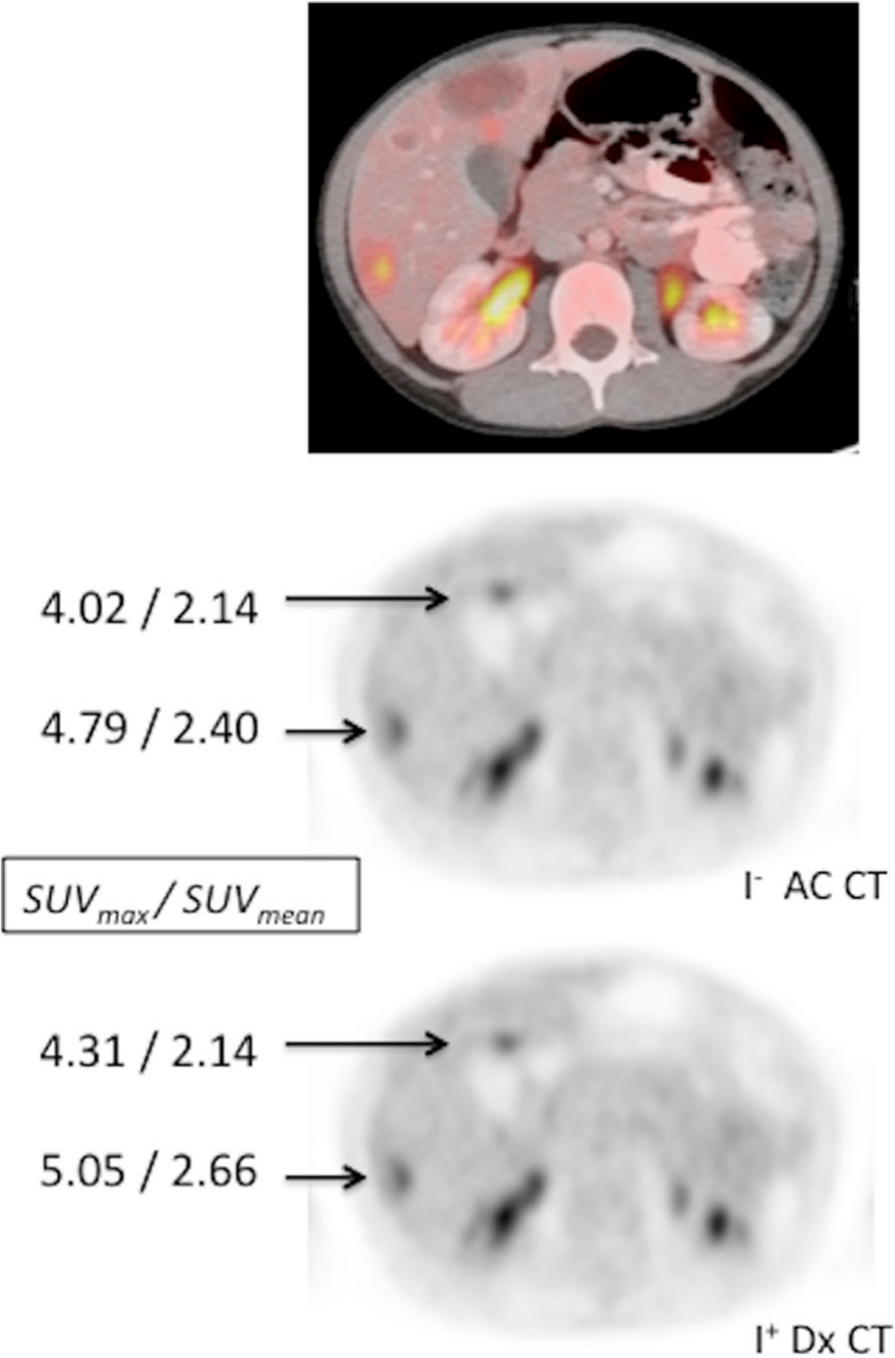
Contrast enhanced Dx CT for PET attenuation has negligible impact on SUV. Non-contrast low dose AC CT and contrast enhanced Dx CT were used to perform attenuation correction on ^18^F-FDG PET images from a 6 yo child being treated for Burkitt lymphoma. A representative PET image of the liver, fused to the accompanying Dx CT, shows multiple FDG-avid liver lesions. PET images obtained following attenuation correction using the non-contrast AC CT and the contrast enhanced Dx CT show negligible differences in SUV values (SUV_max_/SUV_mean_) calculated for representative liver lesions

**Fig. 9:**
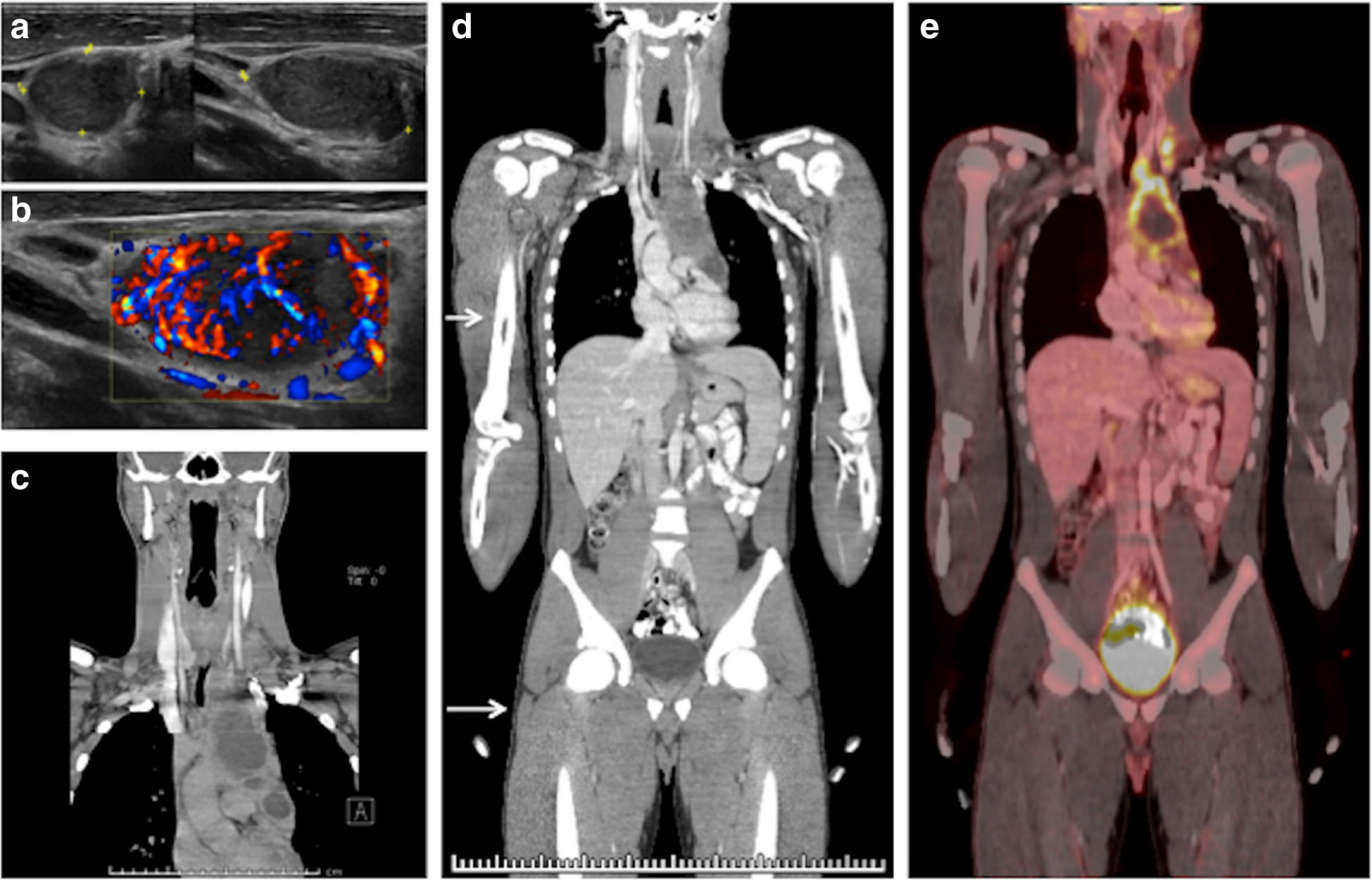
Multi-series PET/CT with Dx Abdomen/Pelvis CT. Eighteen years old patient with cervical adenopathy was evaluated initially by US (**a**, **b**). Subsequent evaluation included a diagnostic CT of the neck and chest (**c**). Biopsy revealed Hodgkin lymphoma. Completion staging involved a diagnostic CT of the abdomen and pelvis, which was incorporated into the PET/CT acquisition (**d**, **e**), to avoid repeat diagnostic CT imaging of the neck/chest and double scanning of the abdomen and pelvis. Arrows (**d**) show the junction between low dose AC CT and diagnostic portions of the exam

To address these concerns, others have shown [26, 27], and we have verified (Fig. 8) that IV contrast does not significantly impact either SUV max or SUV mean calculations. We then worked with our PET/CT manufacturer (Siemens Healthineers, Hoffman Estates, IL, USA) to develop and validate the acquisition software to allow for obtaining a multi-series CT acquisition as part of the PET/CT exam, such that the patient receives only the dose required for each area, with minimal overlap. For example, as shown in Fig. 9, a non-diagnostic attenuation correction scan can be acquired from the skull base through the thorax, followed by a diagnostic scan of the abdomen and pelvis, finishing with a non-diagnostic low dose scan to the mid-thighs to match the remaining PET bed position. These separate series are then merged into a single data set, which can be used for attenuation correction, anatomical correlation and diagnostic interpretation. This multi-series PET/CT approach is now routine for the majority of our PET/CT acquisitions that require both diagnostic and non-diagnostic CT exams.

Tables 1 and 2 provide details for the attenuation correction and diagnostic CT parameters used during PET/CT exams.

**Table 1:** Protocol Details – I^+^ low dose AC CT

• Low dose I^+^ enhanced optimized AC CT
- 2 cc/kg IV contrast (Optiray320); 1 cc/kg if <10 kg; 22 G - 2 cc/s injection, 10–20 cc saline flush, scan - No oral contrast routinely used - Arms down, elevated off table with blanket • (arms up possible, depending on pt)
• CT scan top - > down (eyes - > thighs, vertex-toes)
• Thickness: 1.2 mm, Pitch: 1.3 • Tube current modulation (reference mAs, weight-based): 20 mAs (< 55 kg); 35 mAs (> 55 kg) - Reconstructions: • Sag, Cor: standard algorithm • Axial: lung algorithm • Iterative Reconstructions: 2 iterations

**Table 2:** Protocol Details – Dx CT for Attenuation Correction

• Standard dose po/I^+^ contrast enhanced Dx CT
- 2 cc/kg IV contrast (Optiray320); 1 cc/kg if <10 kg - 2 cc/s injection, 10-20 cc saline flush - Oral contrast (1 cc Opti 320/30 cc water, max 300 cc) • Adjustment for sedation (60 min delay after last cup) - Arms down, elevated off table with blanket • (arms up is possible, depending on pt)
• CT scan top - > down (eyes - > thighs, vertex-toes)
• Thickness: 0.5 mm • Pitch: 1.3 – Tube current modulation/reconstructions: standard Dx CT – Dedication lung CT, end-inspiration, as dedicated

As described earlier in the “Ordering the Examination” section, and shown diagrammatically in Fig. 3, the following imaging algorithm is now routinely used:

1. Determine whether the PET/CT examination is to be done with or without a diagnostic CT
2. Determine what the PET coverage will be (Torso, whole body, or limited).
3. For PET/CT without a Dx CT, follow the approach outlined in Table 1, with weight based tube current modulation. We routinely use IV contrast for the attenuation correction CT, with a dose of 2 cc/kg and a rate of 2 cc/sec. Axial, sagittal and coronal reconstructions are generated, in addition to axial reconstructions using a lung algorithm.
4. For a PET/CT with a Dx CT, the ordering clinician must specify the CT coverage. Regardless of the Dx CT coverage, the patient preparation (IV, oral contrast), weight-based reference mAs, pitch and reconstruction techniques should match the standard Dx CT protocols in use on other CT scanners in the department. These are summarized in Table 2.

**Fig. 10:**
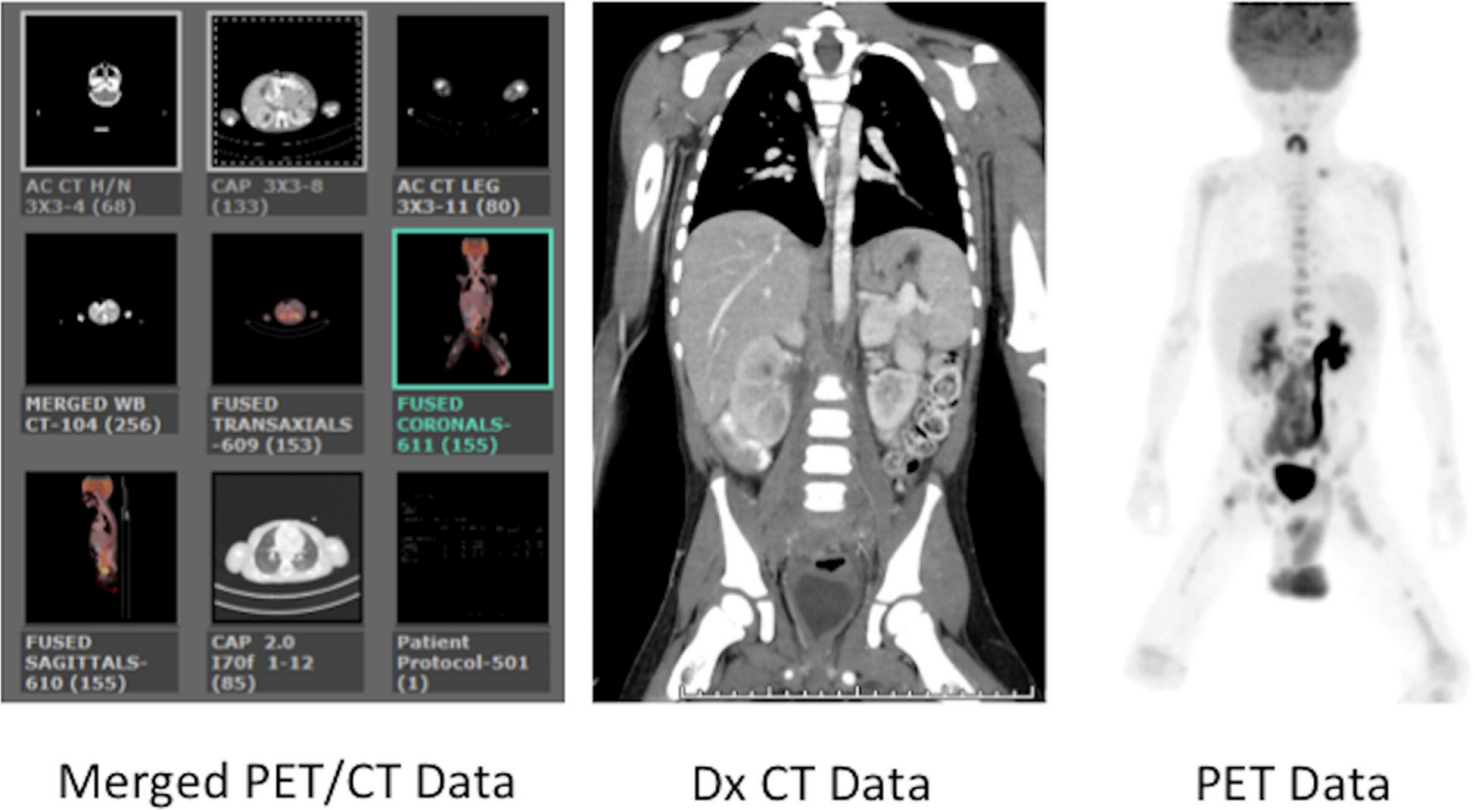
Multi-series PET/CT. Screen capture from the acquisition workstation shows the components of the multi-series PET/CT exam, in this case including low dose AC CT coverage of the Head/Neck, Dx CT coverage of the Chest, Abdomen, and Pelvis, and completion low dose AC CT coverage of the upper legs to match the PET acquisition. The merged CT data used to attenuation correct the PET data is displayed as a separate series at the workstation. Images from both the PET/CT and the diagnostic CT examinations are sent to their respective PACS exam folders and reported separately

Based on the Dx CT coverage, one of three acquisition techniques are used:

- PET/CT with a separate Dx CT (for example, an non-contrast Chest CT to evaluate for lung nodules is performed separately from – usually before – the PET/CT exam given the need for end-inspiration CT imaging and no need for IV contrast for the diagnostic CT exam).
- PET/CT with a Dx CT of the same coverage (Torso CT is used both for diagnostic interpretation and PET attenuation correction). This scenario is common for lymphoma patients.
- PET/CT with a Dx CT limited to a specific region of interest that can be readily incorporated into the PET examination. This scenario is common for lymphoma patients who may have had a Dx neck and chest CT at the time of initial presentation. Once the diagnosis is confirmed, an Abdomen/Pelvis CT is required for completion of staging, together with a torso PET. In this scenario, the examination is performed in 3 series: a low dose Neck/Chest series, a diagnostic Abdomen/Pelvis series, and a small low dose series of the extremities to match the PET bed positions. As described earlier, these 3 series are merged into a single data set and used for attenuation correction, fusion/anatomic co-localization with the PET, and diagnostic interpretation (Fig. 10).

PET/CT acquisition techniques vary considerably between institutions [5]. One result of implementing our multi-series acquisition technique has been a reduction in radiation dose at our institution. In phantom experiments, Fahey et al. showed that dose reductions of as much as 44% can be achieved using an integrated multi-series PET/CT acquisition technique, as compared to 2 separately acquired examinations [5]. In addition to reducing unnecessary radiation dose, having diagnostic quality CT data for image co-registration and fusion can also improve the diagnostic accuracy of the PET examination and provide anatomic correlation for lesions that would have been difficult to discern on a low dose attenuation correction CT.

In all cases, the CT used for attenuation correction, whether low dose or of diagnostic quality, should be acquired with the same patient positioning and the same quiet breathing used for the PET acquisition. This is particularly important for lesions in the chest and upper abdomen, where large differences in patterns of breathing (e.g. end-inspiration vs quiet breathing) can lead to mis-registration artifacts [22]. If an end-inspiratory diagnostic quality chest CT is required as part of diagnostic CT torso exam, we will often obtain this separately at the end of the PET/CT examination. Sedated patients will have similar quiet breathing patterns for both the CT and PET acquisitions and co-registered images can usually be accurately generated without difficulty. We do not routinely intubate patients for the purposes of breath-holding during PET/CT.

#### PET acquisition

PET imaging is performed using standard techniques in accordance with the North American and EANM consensus guidelines and the 2016 update of the North American guidelines [28, 29], using administered activities of 3.7-5.2 MBq/kg (0.10-0.14 mCi/kg) for ^18^F-FDG, resulting in effective doses ranging from 5.2-7.4 mSv per examination. Lower administered activities have also been used in an effort to generate sub-mSv PET examinations [30], however these usually require longer acquisition times and have not been rigorously validated to ensure sensitivities and specificities that are comparable with the existing techniques.

Patients are placed in a warm injection room (~24 °C/75 ° F) for at least 30 min prior to FDG injection to reduce FDG uptake in brown adipose tissue. Others have reported using β-blockers, such as propranolol, low dose benzodiazepine (diazepam), or short-acting opiates such as intravenous fentanyl in an effort to reduce brown fat uptake [22]. In our experience, proper patient preparation, with instructions to avoid cold exposure and dress warmly (even during warm summer months, ambient temperatures in air conditioned cars and hospitals may be quite cold for a lightly dressed child), followed by warming of the patient prior to, and during the uptake period, can substantially eliminate brown fat uptake in most patients. We do not routinely administer benzodiazepines, opiates, or other pharmaceuticals during the PET/CT examination, and in many institutions use of these agents is considered procedural sedation and requires prior consultation with anesthesia and/or sedation services. During the uptake period patients are also instructed to minimize repetitive muscle activity in an effort to reduce background muscle uptake, although in practice the patterns of muscle uptake in children related to, for example, inconsolable crying, use of a pacifier, or use of electronic devices such as cell phones, can usually be readily recognized and interpreted.

Following FDG injection, an uptake period of 60 min is standard for most body imaging and oncologic applications. For brain tumor imaging, a shorter uptake period can be utilized (30 min) but should be standardized for all such studies. The PET/CT acquisition is ideally started as soon as possible after the 60 min tracer uptake period. Wide variability in uptake times can affect the reproducibility and comparability of SUV values between studies, although in practice it is challenging – particularly for sedated patients – to adhere rigorously to the 60 min uptake period guideline, and it is generally accepted that absolute SUV values are prone to 10-15% variability, related both to unavoidable differences in uptake time and variability in tissue biodistribution between examinations [31].

The PET/CT acquisition and reconstruction parameters will depend on the equipment available. On our Siemens mCT 40 PET/CT with a 20-cm axial field of view, PET images are acquired in 3D mode using an acquisition time of 3 min per bed position (Table 3), with the acquisition proceeding from head to toe. Reversing the direction of the PET acquisition, moving from feet to head, may be indicated, particularly for bladder and pelvic neoplasms where excreted tracer in the bladder can obscure tumor uptake, although as noted earlier, placement of a bladder catheter could be considered in such circumstances. Arms may be positioned above the head or be placed at the patient’s side, depending on the indication for the exam. Head and neck tumors are best imaged with the arms down to minimize CT beam-hardening artifacts in the neck. Similarly, tumors in the thorax and upper abdomen may benefit from having the arms up, although young children often find it difficult to hold their arms above the head for the ~20 min PET acquisition without some additional support (handles or the head holders supplied by most manufacturers). In practice, having the arms at the patient’s side, slightly elevated off the bed to reduce the beam hardening that can occur when upper arms and the vertebral column are in alignment, is adequate for most pediatric indications, and preferred for sedated patients in whom having the arms above the head may compromise the airway and IV access.

**Table 3:** Protocol Details – ^18^F-FDG PET

• Either I^−^ or I^+^ low dose-optimized AC CT or Dx CT is obtained prior to PET imaging
• PET – ^18^F-FDG
– 150 uCi/kg (5.55 MBq) • min: 500uCi (18.5 MBq) • max: 10 mCi (370 MBq) – Arms down, elevated off table with blanket • (arms up is possible, depending on pt) – PET imaging top - > bottom (eyes- > thighs, vertex-toes) – 3 min/bed position (20-30 min), depending on size

Iterative reconstruction of the PET data has been standard for more than 15 years. However, the reconstruction of the 3D PET data will require either a 3D reconstruction algorithm or rebinning of the data into a 2D set prior to reconstruction. Many sites utilize ordered subset expectation maximization (OSEM) iterative reconstruction leading to a reduction in reconstruction time. Lastly, we routinely use iterative reconstruction with resolution recovery which has improved the imaging of small structures, particularly in our younger patients.

### PACS integration

Once the PET/CT examination is complete, attenuation correction of the PET imaging data and co-registration with the previously determined CT dataset is performed at the acquisition workstation. For routine examinations done without a diagnostic CT, the PET and attenuation correction CT exams are automatically processed and sent to the Picture Archiving and Communication System (PACS). When a diagnostic CT is being integrated into the PET/CT exam, the respective low dose and diagnostic CT series are first merged, followed by attenuation correction of, and co-registration to, the PET data.

When sending the PET/CT data to the PACS system, at a minimum the following series should be sent with every examination: 1) the attenuation-corrected PET exam, 2) the non-attenuation corrected PET data, and 3) the co-registered CT images used for attenuation correction. Inclusion of the uncorrected PET data is important to allow apparent focal areas of FDG uptake that are related to attenuation correction artifacts, rather than disease, to be evaluated. Additional series that we routinely include with all PET/CT examinations include a maximum-intensity-projection (MIP) image of the PET examination, axial, sagittal, and coronal fused PET/CT images, axial, sagittal, and coronal CT images reconstructed using a soft-tissue reconstruction kernel, and axial images of the thorax processed using a lung technique.

When a Dx CT has been integrated into the PET/CT examination, special considerations are needed to allow the diagnostic CT images to reside simultaneously in the PACS system within both the PET/CT folder and the Dx CT folder. Most PACS systems prevent the same imaging data from being sent to two different destinations within a given patient’s examination folder by assigning each acquisition series a unique ID or UID. This is to prevent the inadvertent placement of duplicate exams in the patient’s image archive, which can result in confusion and potentially lead to erroneous image interpretation. Depending on both the PET/CT platform and the PACS vendor, a protocol must be developed to allow the diagnostic CT data to co-exist within the PET/CT exam folder, either separately or as part of a merged CT dataset, and within the diagnostic CT folder.

For example, using the scenario described previously, in which a patient undergoes a torso PET with a Dx CT of the Abdomen/Pelvis, the following workflow is used:

1. Both the PET/CT Torso and the Abdomen/Pelvis Dx CT exam orders are activated, which assigns PACS acquisition numbers to each of the orders.
2. Once the PET/CT is complete, the PET examination is processed using the merged CT data, as detailed above. When the processing of the PET/CT exam is complete and the images have been sent to PACS, the Dx CT portion of the PET/CT examination is transferred into the Dx CT folder on the acquisition workstation (this folder will only be available if the both the diagnostic CT and PET/CT orders have been activated prior to the exam) and then sent to PACS. Other strategies are possible, but we have found this to be an efficient workflow that minimizes procedural errors during the image transfer process.
  It is also the practice in many nuclear medicine departments to send PET/CT images to a dedicated nuclear medicine viewing workstation to afford a more comprehensive review of the PET/CT data. At our institution, images are reviewed using the Hermes GOLD™ (Hermes Medical Solutions) viewing platform, although other vendors such as MIM Software, Inc. provide similar functionality. Most PACS vendors also include some type of PET/CT viewing capability within the PACS environment (e.g. Synapse 3-D, GE Centricity Universal Viewer, etc.), which may be adequate, depending on the clinical environment.
3. Once the examination has been completely processed and delivered to the appropriate PACS folders, the technologist performing the examination should confirm receipt of the correct images in PACS and assure that both the diagnostic CT and PET/CT folders have the appropriate images needed for interpretation. Separate dictations are then issued for each of the respective examinations.

### PET/CT interpretation

#### Interpretation

Each department will have its own preferred workflow for interpreting PET/CT examinations, although in practice most radiologists and nuclear medicine physicians review these complex examinations similarly.

All PET images should be reviewed in the transverse/axial, coronal and sagittal planes, reviewing both the fused and un-fused data. This is best accomplished using either a dedicated nuclear medicine processing and viewing workstation or a PACS-integrated nuclear medicine viewing functionality. Regardless of the viewing environment chosen, it is essential that PET image thresholds be scalable and adjustable. The fusion workstation should have the capability of displaying fused images with different percentages of PET and CT blending, and should have the capability of measuring SUV, including use of volumetric ROI.

We generally review the PET component of the exam first, using the greyscale images on an appropriately calibrated monitor. Beneath the PET images, the fused images are displayed, which allows for convenient anatomic co-localization of any PET abnormalities. The fused images are then separately reviewed in all three planes, followed by a dedicated review of the CT images in all three planes (whether low dose attenuation correction CT, diagnostic CT or merged hybrid CT images).

The CT images are also reviewed at a diagnostic PACS workstation in all three planes, using soft tissue, bone and lung windows, to evaluate for incidental/unexpected findings. If a Dx CT scan was requested and performed as part of the PET/CT examination, then the Dx CT should be reported separately to remain compliant with regulatory and reimbursement requirements. The physicians interpreting the PET/CT or the Dx CT must satisfy institutional credentialing requirements, and in our practice the radiologist interpreting the diagnostic CT is usually separate from the physician interpreting the PET/CT. Although is it not standard practice in most institutions, we issue a separate report for the attenuation correction CT portion of the exam, making reference to any pertinent findings on the accompanying PET examination, and ensuring a thorough and comprehensive review of all the available imaging data.

#### Report generation

In general, three reports are provided for each examination: the PET report, the attenuation correction CT report and, when applicable, a diagnostic CT report.

##### PET report

In accordance with ACR and SNMMI practice guidelines, the PET report should contain a description of the radiopharmaceutical used, the administered activity and route of administration, serum glucose level, patient weight and time of injection. Since this will be the primary report reviewed by the referring clinician, any additional information, such as need for sedation or contrast reactions, should be noted.

Description of any areas of abnormal FDG uptake should be noted, with relation made to any correlative findings on the CT images and with provision of any quantitative or semi-quantitative measures of FDG accumulation (SUV). Any image artifacts or technical problems that could lead to image misinterpretation should also be noted.

##### Attenuation correction CT

Although not standard practice or required by the guidelines, it is our practice to review the attenuation correction CT images with the same rigor used for reviewing a diagnostic CT. A standardized reporting template is used, with brief descriptions of any CT findings that correlate with PET abnormalities, and note made of any pertinent incidental or unexpected findings (e.g. spondylolysis, vertebral compression, malpositioned support lines or catheters, etc).

##### Diagnostic CT

This is reported by the covering body radiologist using standard dictation templates, in a similar fashion to a Dx CT being obtained without an accompanying PET examination. In most cases the radiologist reviews the Dx CT together with the individual interpreting the PET examination, which insures a comprehensive review and allows both the PET and Dx CT reports to convey uniform information.

### Artifacts

A discussion of the numerous artifacts that one may encounter during PET/CT examinations is beyond the scope of this review and has been described previously [22]. Where indicated strategies for minimizing specific artifacts or physiologic variants (i.e. muscle and brown fat uptake) have been noted elsewhere throughout this review.

## Conclusions

PET/CT plays an important role in the management of many pediatric malignancies. This review provides an overview of our approach to determining when and how a PET/CT examination is performed, with attention to optimizing the CT component of the PET/CT exam. A multi-disciplinary approach is needed to ensure that the indications for PET/CT are appropriate, that the correct examination is performed, and the appropriate imaging techniques are used. As hybrid imaging technologies such as PET/CT have evolved, new strategies for image acquisition and interpretation have emerged, leading to many exciting opportunities for improving the overall quality of the PET/CT experience.
